# The First Study of Mating Mistakes in Stoneflies (Plecoptera) from China, with Remarks on Their Biological Implications

**DOI:** 10.3390/insects13121102

**Published:** 2022-11-30

**Authors:** Qing-Bo Huo, Bin-Qing Zhu, Dávid Murányi, José Manuel Tierno de Figueroa, Meng-Yuan Zhao, Ya-Nan Xiang, Yu-Ben Yang, Yu-Zhou Du

**Affiliations:** 1 School of Horticulture and Plant Protection & Institute of Applied Entomology, Yangzhou University, Yangzhou 225009, China; 2 Department of Zoology, Eszterházy Károly Catholic University, Leányka u. 6, H-3300 Eger, Hungary; 3 Nanjing Institute of Environmental Sciences, Ministry of Ecology and Environment, Nanjing 210042, China; 4 Departamento de Zoología, Facultad de Ciencias, Universidad de Granada, Campus Fuentenueva s/n, 18071 Granada, Spain; 5 Joint International Research Laboratory of Agriculture and Agri-Product Safety, the Ministry of Education, Yangzhou University, Yangzhou 225009, China

**Keywords:** Perlidae, Perlodidae, Styloperlidae, biology, behavior, competition

## Abstract

**Simple Summary:**

Adults of stoneflies have diverse mating behaviors and complex signals for communication, but they are not always able to correctly recognize their mates. With the observations from several provinces of China, we provide the first study on the erroneous mating behaviors of stoneflies from this country. Three different categories of erroneous mating attempts involving 13 species belonging to three stonefly families are reported, and information on their physical competition, the sensorial mechanisms triggering the mating, the conditions favoring the mating mistakes, and the possible consequences of interspecific mating are discussed. Hitting and pushing with the head and abdomen could be the unique method employed in the male–male physical competition. Vibrational signals are considered not a prerequisite for triggering a mating behavior, while vision and/or touch could be a sufficient condition for triggering it, but they are not always efficient for species-specific recognition.

**Abstract:**

Currently, information on the biology of Plecoptera from China is scarce, particularly on mating behavior. In this paper, the existence of mating mistakes (erroneous mating attempts) involving 13 Chinese stonefly species (belonging to nine genera and three families) is reported. These erroneous mating behaviors can be included into three different categories: mating attempts between conspecific males (including the formation of erroneous mating balls), mating attempts between different taxa (including displacement attempts during copulation), and mating-related behaviors with non-living objects. From these behaviors, some aspects of stoneflies during mating, such as the physical competition between males, the sensorial mechanisms implied in triggering a mating behavior, the conditions favoring the mating mistakes, and the possible consequences of interspecific mating in the hybrid production, are discussed.

## 1. Introduction

In the mating of Plecoptera (stonefly), male and female adults exhibit diverse behaviors, some of them not yet well known [1]. More or less complex vibrational signals (mainly produced by drumming, but also by rubbing, tremulation, or a combination of them) are considered to be the main mechanism for mate recognition and encounter in stoneflies [2,3,4], but the use of these signals is not an indispensable prerequisite for mating [1,5]. Other mechanisms as the existence of encounter sites can play an important role in aggregating sexes [1,6] and, probably, the employment of other intersexual communication channels (tactile, visual, olfactory) could be also useful for the recognition [1,4,7]. Nevertheless, adults, particularly males, are sometimes poorly selective, and they can attempt to mate with other conspecific males, different species, or even dead individuals [8,9,10,11,12].

The mating behavior of stoneflies has been documented since the past century mostly in Europe and North America, but in China, the existing studies on Plecoptera are mostly taxonomic [13,14,15,16,17,18], and there is poor knowledge of the stonefly biology, especially on mating behavior, of which only the drumming signal of only one species has been recorded [19].

Recently, we observed multiple cases of erroneous mating behaviors in stoneflies from China (observed in natural and artificial environments). The aim of this study is to report erroneous mating behaviors document for Chinese species for the first time.

## 2. Materials and Methods

This study was conducted without harming protected or endangered species, and all research activities were authorized. In the process of observation and recording, all kinds of artificial optical and sound interference were excluded as much as possible, except the street lights nearby the stream, the noise of passing vehicles, and the flash of the camera.

The specimens observed and studied were collected in the Anhui, Fujian, Shaanxi, and Qinghai provinces of China. The exact localities are detailed in Table 1, Table 2 and Table 3. All observations were carried out from April 2020 to July 2021. Some behaviors were detected directly in the field with specimens on natural substrates, while in other cases specimens collected by hand or light traps were observed inside the dry, clean and empty (sometimes with few tissue paper) bottles (size: ca. 220 mm high, 500 mL, or 400 mm high, 1500 mL) or plastic boxes (size: 290 × 190 × 100 mm) used to take them to the laboratory. Soft crumpled paper was used into a mass for stuffing into the narrow bottleneck when adult stoneflies were collected and introduced inside the bottles.

After recording the observations in natural or artificial substrates, all specimens were transferred to the laboratory and preserved in 75% ethanol. In the laboratory, the identification of specimens was confirmed under a KEYENCE VHX-5000 system. Photos were taken with Canon cameras (EOS 5D Mark IV, PowerShot SX730 HS) and optimized by Adobe Photoshop CS6. The materials are deposited in the Insect Collection of Yangzhou University (ICYZU).

## 3. Results

Erroneous mating attempts involving three families, nine genera, and thirteen species of stoneflies were recorded (Table 1, Table 2 and Table 3). These behaviors can be included into three different categories: mating attempts between conspecific males (including the formation of erroneous mating balls), mating attempts between different taxa (including displacement attempts during copulation), and mating-related behaviors with non-living objects.

### 3.1. Mating Attempts between Conspecific Males

Mating attempts between intraspecific males have been observed in three Perlidae species (*Claassenia magna* Wu, 1948, *Claassenia* sp., *Togoperla tricolor* Klapálek, 1921) and only one Perlodidae species, *Perlodinella epiproctalis* (Zwick, 1997) (see Table 1). These erroneous mating behaviors of *Claassenia magna* and *Perlodinella epiproctalis* only occurred in artificial environments (plastic box or bottle).

**Figure 1:**
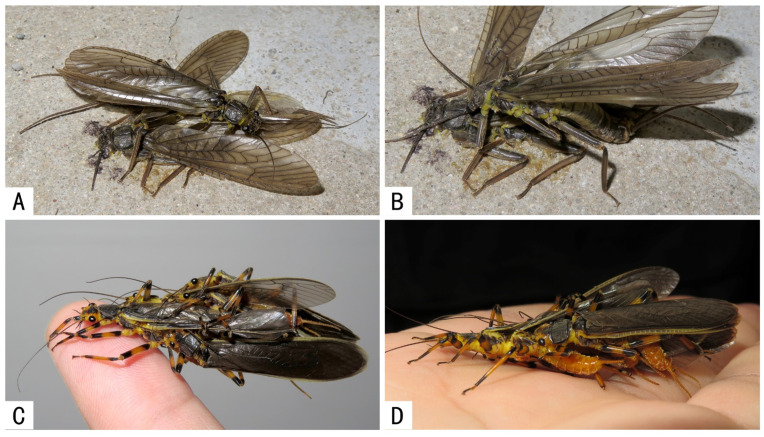
Intraspecific male–male erroneous mating attempts. (**A**,**B**) *Claassenia* sp., the male attempting to mate with a dead male; (**C**,**D**) *Togoperla tricolor*, erroneous mating ball made up by only males, which use their abdomens to push each other.

In addition, *Claassenia* sp. from the Qinling Mountains of Shaanxi were observed attempting to mate with a conspecific dead male (Figure 1A,B), while *Togoperla tricolor* from Fujian were recorded forming erroneous mating balls composed of multiple males but without any females (Figure 1C,D).

### 3.2. Mating Attempts between Different Taxa

**Figure 2:**
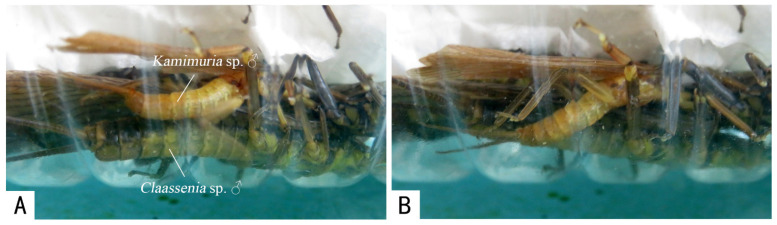
A male of *Kamimuria* sp. climbing on a male of *Claassenia* sp. from the lateral side (**A**) and attempting to mate (**B**).

**Figure 3:**
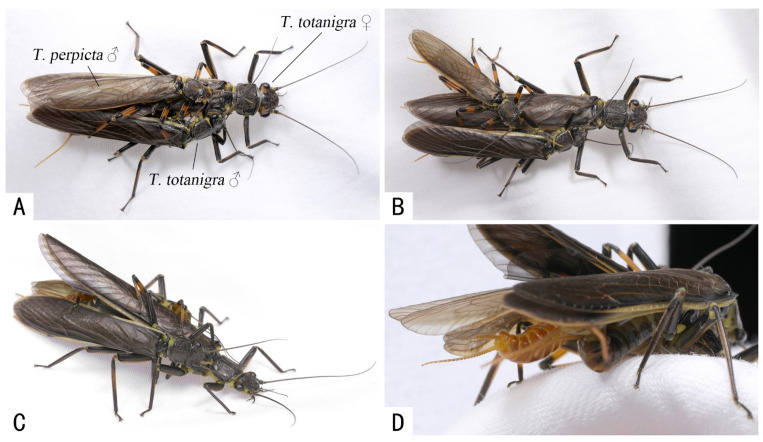
Male competition between *Togoperla perpicta* and *Togoperla totanigra*. (**A**,**B**) *T. perpicta* trying to push the *T. totanigra* male with head; (**C**,**D**) *T. perpicta* changes its position, then uses his abdomen to beat the abdomen of *T. totanigra* male.

Erroneous mating attempts between different species, genera, or families, involving seven genera and nine species of Perlidae (*Flavoperla biocellata* Chu, 1929, *Hemacroneuria violacea* Enderlein, 1909, *Kiotina* sp., *Claassenia* sp., *Kamimuria tienmushanensis* Wu, 1938, *Kamimuria* sp., *Neoperla sinensis* Chu, 1928, *Togoperla perpicta* Klapálek, 1921, *Togoperla totanigra* Du & Chou, 1999) and one genus and species of Styloperlidae (*Styloperla inae* Chao, 1947) were recorded (see Table 2, Figure 2 and Figure 3).

All the cases involving *Flavoperla biocellata*, *Neoperla sinensis,* and *Kamimuria* sp. occurred in an artificial environment (plastic box or bottle), and the process often only took from a few seconds to a dozen seconds. Individuals targeted by mating attempts showed strong resistance and always kept running until these males attempting to mate with them were dissuaded.

An outstanding example of mating displacement was noticed in one male *T. perpicta* on a coupled *T. totanigra* pair (Figure 3). During this process, the *T. perpicta* male mildly touched the *T. totanigra* female body with his antennae and mouthparts, while roughly hitting and pushing the *T. totanigra* male with his head and swinging his abdomen from the side, forcing the latter to leave the mating. In this competition case, the mating displacement began when the aedeagus of the *T. totanigra* male was completely inserted into the female genital opening (they were in the copulation phase within the mating sequence), and no previous drumming behavior was observed in the *T. perpicta* male. An accidental shaking of the container where the individuals were during the displacement behavior caused the disturbed *T. perpicta* male to escape to a distance of more than 20 cm from the *T. totanigra* female, but then quickly return, accurately climbing on the female’s back and started again to compete with the *T. totanigra* male until it drove away the male *T. totanigra*.

### 3.3. Mating-Related Behaviors with Non-Living Objects

**Figure 4:**
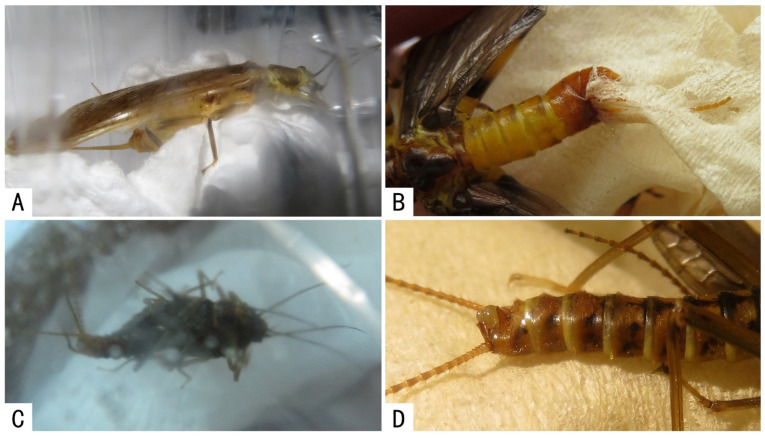
Male stoneflies performing mating-related behaviors with paper material. (**A**) *Claassenia magna* rubbing the abdomen on the paper; (**B**) *Togoperla tricolor* gripping the paper with hemiterga and abdominal process; (**C**) *Perlodinella epiproctalis*, males participating in an erroneous mating attempt with one of them rubbing the abdomen on the paper; (**D**) *Perlodinella epiproctalis* rubbing the abdomen on the paper, with the aedeagus and epiproct partly everted.

Mating-related behaviors of male stoneflies with non-living objects (paper in plastic box/bottles), involving two genera and three species of Perlidae (*Claassenia magna*, *Claassenia* sp., *Togoperla tricolor*) and one genus and species of Perlodidae (*Perlodinella epiproctalis*), were also observed (see Table 3 and Figure 4).

These observations were accidental. Regardless of whether the bottle had previously contained any stoneflies, or whether there were one or more individuals, males were likely to develop an erroneous mating-related behavior with the crumpled tissue or the raised area of the bottle.

## 4. Discussion

Mating behavior consists in all events from the pair formation through the active searching, attraction, and/or courtship to the final separation of the pair [20], or even more, if the fertilization and other postcopulatory behaviors are also included as components of the mating sequence [21]. In any case, mating behavior can be very complex, flexible, and diverse in insects [20].

The existence of mating mistakes or erroneous mating behaviors has been repeatedly reported in the literatures for many different animal groups, including insects, and it is usually considered as a consequence of poor sex, instar or species recognition by males, or scarce selectivity [20,22]. These erroneous mating behaviors are usually associated with fitness loss [23], and many mechanisms have evolved for improving or assuring the correct identification of the mate facilitating erroneous mating avoidance.

In Plecoptera, as reported in the Introduction, the existence of erroneous mating behaviors, including male mating attempts with individuals of different species, with other conspecific males, with dead females or with nymphs, have been reported in different areas, mainly from North America and Europe, both in nature and in the laboratory [1]. Nevertheless, the information on this topic is sparse and no data exist for China, a highly diverse stonefly region. Our results contribute to solving this lack of information and show that erroneous mating attempts can be more common than usually reported. They also shed some light on male–male competition aspects during mating.

When describing the formation of mating balls in stoneflies, Tierno de Figueroa et al. [24] noted that, as pointed out by Shine et al. [25] in other animal groups, mating balls are most likely to occur in species of animals that do not display male–male combat. The inexistence of combat does not mean that during the mating ball each male does not attempt to displace the others (a way of male–male competition) pushing them and attempting to copulate with the female. Attempts in which a male climbs on a copulating pair and attempts to mate with the female and displaces the original male have also been reported in stoneflies [5,26]. In our study, the behavior of *Togoperla tricolor* (during an erroneous mating ball) and of *T. perpicta* (involved in a male displacement acting on a *T. totanigra* couple) confirms that male stoneflies carry out intense physical conflicts when competing for mates, like many other animals. It is well known that the body of stoneflies is mostly soft and they lack hypertrophied mandibles or horns that can act as spectacular weapons [27], such as in the case of some beetles and dobsonflies. Previous observations during mating displacement attempts showed that hitting and pushing with the abdomen seems to be the only way stoneflies use for attempting to dislodge other males but with poor success [5,26], so it is particularly interesting that head-butting also affected the displacement by *T. perpicta*.

The erroneous mating attempts detected in the present study, being particularly significant in the case of *Claassenia* sp. males attempting to mate with a dead individual, confirm that the use of vibrational signals is not a prerequisite for triggering a mating behavior [1,5].

Our study suggests that vision and/or touch could play a key role in most cases and be a sufficient condition for triggering a mating behavior, but they are not always efficient for species-specific recognition. The examples of mating-related behaviors with non-living objects, particularly in cases in which the male was alone and no other stoneflies have been previously there leaving any odorous molecule, seem to coincide with the fact that to date no studies have shown the existence of identification contact sex pheromones in Plecoptera [28]. Nevertheless, studies on this topic in stoneflies are lacking, and a recent electrophysiological study [29] has shown that adult stoneflies can perceive olfactory cues through their antennal sensilla. Therefore, new studies focused on the possible existence of sexual pheromones in stoneflies are required.

Although in stoneflies the use of species-specific vibrational signals is recognized as a very important mechanism for mate encounter and recognition [1,4,6,30], sometimes, when adults coincide on a relatively small surface, the vibrational signals can be omitted or they are unsuccessful in avoiding interspecies mating, not preventing a male from trying to copulate with an individual that did not answer to his drumming call.

It is known that in small areas, particularly in artificial substrates where individuals are at high densities and are forced to be in close contact, the frequency of interspecific mating in insects is higher [23]. This could explain part of our observations, although erroneous mating attempts in stoneflies have been detected in our study also in natural conditions. In fact, mating mistakes in insects are not uncommon despite that they are seldom successful in nature [28]. Nevertheless, both hybridization and mitochondrial introgression have been reported in stoneflies [12,31,32]. Unfortunately, we do not know how common these mating mistakes are in nature in different stonefly species, and whether they can produce fertile filial generation, even leading to local sympatric speciation. As Abbott et al. [33] stated, hybridization “may provide the raw material for adaptive divergence or initiate new hybrid populations, potentially leading to speciation”. The results of these interspecific crossings and the characteristics of their progeny (if any) would need to be further identified and analyzed, but it is a complex task. It is difficult to confirm the existence of hybrid individuals in nature from a few specimens and based especially on morphology. Recently, some stoneflies from China showing significant intraspecific morphological variability [34,35,36] and genetic differences have been reported [37], but further collecting and sequencing studies are still required.

In fact, the consequences of the reticulation events (including hybridization and introgression) [38] in some other insect groups are being increasingly studied, particularly since hybridization has been recognized to play an important role in the evolution and adaptation not only of the plants but also of the animals [38,39]. Moreover, changes in the species distribution and phenology as a consequence of the current climate change and other anthropogenic alterations (habitat destruction, pollution, introduction of exotic species, etc.) will probably increase the cases of hybridization and introgression by bringing into contact species that were previously separated spatially or temporally [40,41]. Nevertheless, the consequences of these cases are not clear and, although it could be expected a positive increase in genetic diversity, some negative effects (such as species fusion or threat to the genetic integrity of uncommon species by repeated introgression from the common ones) could occur in the long term [42].

## Figures and Tables

**Table 1:** List of species recorded in erroneous mating attempts with conspecific males.

Individual Attempting to Mate	Individual Targeted by Mating Attempts	Observation Data
*Claassenia magna* ♂	*Claassenia magna* ♂	Fujian: Wuyishan, 2021-V
*Claassenia* sp. ♂	*Claassenia* sp. ♂ (alive/dead)	Shaanxi: Xi’an, 2021-V
*Perlodinella epiproctalis* ♂	*Perlodinella epiproctalis* ♂	Qinghai: Menyuan, 2021-VII
*Togoperla tricolor* ♂	*Togoperla tricolor* ♂	Fujian: Wuyishan, 2021-V

**Table 2:** List of species involved in erroneous mating attempts between different taxa.

Individual Attempting to Mate	Individual Targeted by Mating Attempts	Observation Data
*Flavoperla biocellata* ♂	*Kiotina* sp. ♀	Fujian: Wuyishan, 2021-V
*Kamimuria* sp. ♂	*Claassenia* sp. ♂	Shaanxi: Xi’an, 2021-V
*Neoperla sinensis* ♂	*Hemacroneuria violacea* ♂♀	Fujian: Wuyishan, 2021-IV
*Neoperla sinensis* ♂	*Kamimuria tienmushanensis* ♂	Fujian: Wuyishan, 2021-IV
*Neoperla sinensis* ♂	*Styloperla inae* ♂	Fujian: Wuyishan, 2021-V
*Togoperla perpicta* ♂	*Togoperla totanigra* ♀	Anhui: Huangshan, 2020-IV

**Table 3:** List for the species performing mating-related behaviors with non-living objects.

Individual Performing Mating-Related Behaviors	Non-Living Objects	Observation Data
*Claassenia magna* ♂	Tissue paper	Fujian: Wuyishan, 2021-V
*Claassenia* sp. ♂	Tissue paper	Shaanxi: Xi’an, 2021-V
*Perlodinella epiproctalis* ♂	Tissue paper; bottle	Qinghai: Menyuan, 2021-VII
*Togoperla tricolor* ♂	Tissue paper	Fujian: Wuyishan, 2021-V

## Data Availability

The data presented in this study are available on request from the corresponding author.
